# Climate Variability and Dengue Hemorrhagic Fever in Ba Tri District, Ben Tre Province, Vietnam during 2004–2014

**DOI:** 10.3934/publichealth.2016.4.769

**Published:** 2016-09-26

**Authors:** Le Thi Diem Phuong, Tran Thi Tuyet Hanh, Vu Sinh Nam

**Affiliations:** 1 Ben Tre Provincial Center for Preventive Medicine, Ben Tre Province, Vietnam; 2 Hanoi School of Public Health, Hanoi, Vietnam; 3 National Institute of Hygiene and Epidemiology, Vietnam

**Keywords:** climate variability, dengue fever, dengue hemorrhagic fever, Ben Tre Province, Vietnam

## Abstract

**Background:**

Currently, dengue fever/dengue hemorrhagic fever (DF/DHF) is an important public health challenge in many areas, including the Ba Tri District, Ben Tre Province, Vietnam.

**Methods and Aim:**

This study was conducted in 2015 using a retrospective secondary data analysis on monthly data of DF/DHF cases and climate conditions from 2004–2014 in Ba Tri District, which aimed to explore the relationship between DF/DHF and climate variables.

**Results:**

During the period of 2004–2014, there were 5728 reported DF/DHF cases and five deaths. The disease occurred year round, with peaked from May to October and the highest number of cases occurred in June and July. There were strong correlations between monthly DF/DHF cases within that period with average rainfall (*r* = 0.70), humidity (*r* = 0.59), mosquito density (*r* = 0.82), and Breteau index (*r* = 0.81). A moderate association was observed between the monthly average number of DF/DHF cases and the average temperature (*r* = 0.37). The monthly DF/DHF cases were also moderately correlated with the Aedes mosquito density.

**Conclusions and Recommendations:**

Local health authorities need to monitor DF/DHF cases at the beginning of epidemic period, starting from April and to apply timely disease prevention measures to avoid the spreading of the disease in the following months. More vector control efforts should be implemented in March and April, just before the rainy season, which can help to reduce the vector density and the epidemic risk. A larger scale study using national data and for a longer period of time should be undertaken to thoroughly describe the correlation between climate variability and DF/DHF cases as well as for modeling and building projection model for the disease in the coming years. This can play an important role for active prevention of DF/DHF in Vietnam under the impacts of climate change and weather variability.

## 1. Introduction

Dengue fever/dengue hemorrhagic fever (DF/DHF) is an infectious disease, transmitted by *Aedes* mosquito and has become a major public health concern in various countries worldwide. Dengue epidemics were reported throughout the nineteenth and early twentieth centuries and the disease is found in both tropical and sub-tropical regions, predominantly in urban and semi-urban areas in Asian and Latin American countries [1]. Dengue viruses cause an estimated 50 million infections annually, among approximately 3.6 billion people at risk [2]. Dengue fever was first reported in Vietnam in Hanoi and Haiphong cities in 1959, and since then the disease has become endemic throughout the country. In the northern region, epidemics often occur between June to November, whereas in the southern region, cases have been documented year round [3]. During 1998, the largest DF/DHF epidemic in Vietnam since 1987 occurred, with official data recording 234,920 cases and 377 deaths in 56 out of 61 provinces throughout the country. Since then the disease has been an important public health challenge in the country [3].

Ben Tre province is in the Mekong River Delta, which is considered to be the most vulnerable region to climate change and sea level rise in Vietnam [4]. It is an epidemic area and DF/DHF cases occur year round. The numbers of cases usually pick up in the rainy seasons where the *Aedes* densities increase. From 2009 to 2013 there were 16,881 DF/DHF cases reported in the province. In 2014, there were approximately 700 DF/DHF cases reported and one death, in which Ba Tri District had the highest number of cases (226, accounted for 32.4%) and one death. The prevalence of DF/DHF in Ba Tri District in 2014 was 118.3 cases per 100,000 population, which was 2.2 times higher than that of the Ben Tre province (54.8 cases per 100,000 population) and higher than that of the Southern Region of Vietnam (73.2 cases per 100,000 population) [5]. Previous studies have investigated the effect of climate change on DF/DHF transmission [6]. The risk of DF/DHF has been reported to be associated directly or indirectly with seasonal changes in climate [7],[8]. Since water sources in Ba Tri District are quite salty, people usually use water containers to store rain water for drinking and other domestic purposes, which creates favorable condition for *Aedes* mosquito to breed.

Over the last few decades, there has been an increasing interest among scientists on the health impacts of climate change and weather variability. Numerous studies have been done and substantial results achieved mostly in developed countries, and not many quantitative evidences and assessment of the impacts at local levels were provided for the developing countries, including Vietnam. Some studies have shown that *Aedes* mosquitos are very sensitive to environmental conditions, especially temperature, rainfall, and humidity, while other studies did not find the correlation between rainfall and relative humidity with DF/DHF cases [6]. A study in Metro Manila, Philippines showed that DF/DHF was correlated with rainfall (*p* < 0.05) but not with temperature (*p* > 0.05) [9]. A multiple linear regression models were fitted to look for associations between changes in the incidence rate of DF/DHF and climate variability in the warm and humid region of Mexico and the results showed that the incidence rate or risk of infection was higher during El Niño events and in the warm and wet seasons [10].

In Vietnam, a study in the Central Highland showed that the risk of DF/DHF cases increased in rainy seasons when HI (house index), CI (container index) and BI (Breteau index) values of the vector density increased [11]. In 2011, Kien *et al.* published a book chapter, which showed that the change over time of incidences of malaria and the selected water-borne diseases in Vietnam were correlated with main climate variables (temperature and rainfall), and with inter-annual climate variability (the El Niño) [12]. Although being one of the most vulnerable countries to climate change, in Vietnam and in Ben Tre Province in particular, climate change and disaster issues have not been properly integrated into health policies, strategies and plans. This may partly be due to the weak linkages between policy development and scientific evidence and the lack of reliable quantitative and qualitative data. This study was conducted in 2015, which aimed to analyze retrospective secondary monthly data of DF/DHF cases and climate conditions from 2004–2014 in Ba Tri District, Ben Tre Province of Vietnam to explore the relationship between DF/DHF and climate variables.

## 2. Materials and Methods

### 2.1. The Study Area

Located in Indo-China peninsula of the Southeast Asia or the Greater Mekong Sub-region, Vietnam is a densely populated country and has been recognized as one of the most vulnerable countries in the world to the potential impacts of climate change and rising sea levels. Ba Tri District is located in Ben Tre Province, which is in the South of Vietnam having a tropical climate, with regular tropical typhoons, storms, and extensive flooding. The area is strongly influenced by Southwest monsoon, leading to two distinct seasons, being dry and rainy or wet seasons.

### 2.2. Data Sources

Secondary data was extracted from the monthly and annual reports of the Ben Tre Provincial Department of Preventive Medicine, on the number of DF/DHF morbidity and mortality, patients' records, the results of MAC-ELISA test, and virus identification results for Ba Tri District from 2004–2014. The authors also collected secondary data on monthly vector monitoring results of the same period 2004–2014, reported as the density of mature *Ae. aegypti* population (Density index, abbreviated as DI, which is the average number of female *Ae. aegypti* mosquito caught per inspected house), and the Breteau index (BI, number of positive containers per 100 houses inspected). Data on climate variables included series of monthly mean temperatures, monthly maximum and minimum mean temperatures (°C), rainfall (millimeter), relative humidity (percentage) that was used as primary prioritized climate indicators and were collected at meteology station at Ba Tri District. The data were reported as average monthly data for each year during the studied period 2004–2014. The data set was provided by the Ben Tre Provincial Center for Hydro-Meteorological Forecasting.

### 2.3. Variables and Data Analysis

Variables analyzed in this article included: (1) number of DF/DHF cases reported in Ba Tri District by the Ben Tre Provincial Department of Preventive Medicine from 2004 to 2014; (2) the density of mature *Ae. aegypti* population (DI); (3) the Breteau index (BI); (4) monthly mean temperatures, monthly maximum and minimum mean temperatures (°C), rainfall (millimeter), relative humidity (percentage). Data were analyzed applying Statistical Package for Social Scientist software (SPSS) 18.0. This paper uses descriptive statistics and statistical analysis including Chi Square and Correlation (*r* value) and multivariate regression models to explore the correlation between DF/DHF cases, *Aedes* mosquito density, and climate variables.

## 3. Results

**Figure 1.**
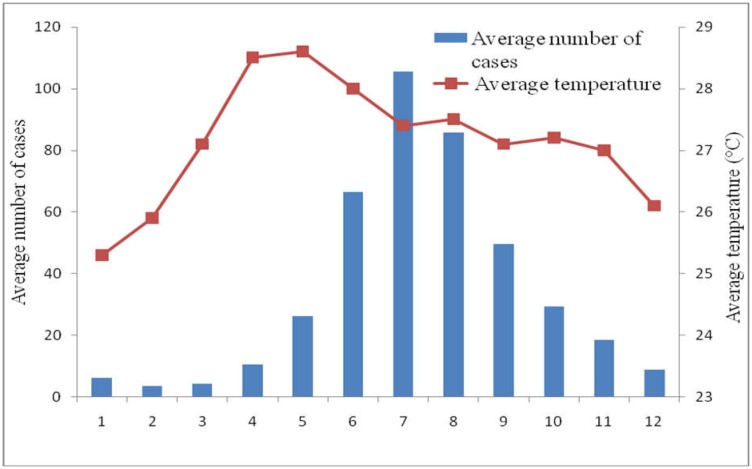
The correlation between DF/DHF cases and average monthly temperature (°C) at Ba Tri District, 2004–2014.

The results showed that the average annual temperature at Ba Tri District was 27.1 °C, and the average temperature was higher (28.6 °C) at the beginning of rainy seasons (May), and then gradually reduced to the lowest level in January and February (26 °C). Average monthly temperature in Ba Tri District from 2004–2014 ranged from 24.5 °C to 30 °C. Generally, the average monthly temperature in the rainy seasons were from 27 °C to 28 °C, which was a favorable condition for *Aedes* mosquito to breed and spread the disease. Figure 1 shows that when the temperature was slightly reduced in the rainy months of June and July, the number of DF/DHF cases started to increase. Additionally, here was approximately a two month lag between the peak temperature reported in May and the peak of DF/DHF occurrences in July. Data during the period 2004 to 2014 showed that there was an average correlation between temperature and DF/DHF cases (*r* = 0.37).

**Figure 2.**
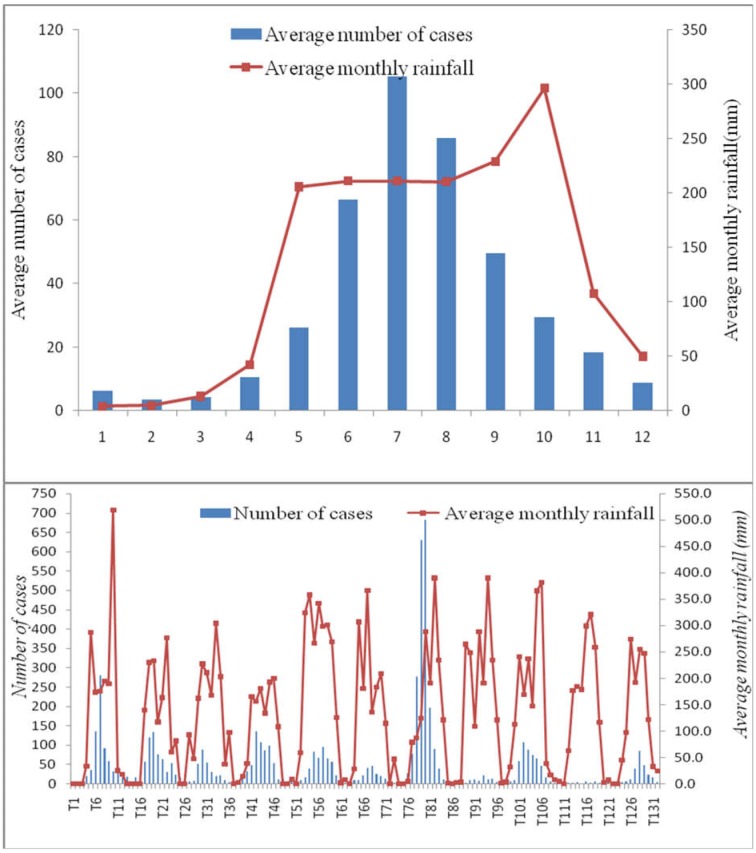
The correlation between DF/DHF cases and average monthly rainfall (mm) at Ba Tri District, 2004–2014.

The average monthly rainfall recorded in Ba Tri District during the period 2004–2014 was 132 mm, with the highest monthly rainfall being 296 mm in July, while there was little or no rain in January and February. The number of DF/DHF cases started to increase from April when rainfall level increased, and continued to increase to achieve the pick level in July before it gradually declined in the following months. The data within the studied period showed a strong correlation between monthly DH/DHF cases and average monthly rainfall (*r* = 0.70, Figure 2).

**Figure 3.**
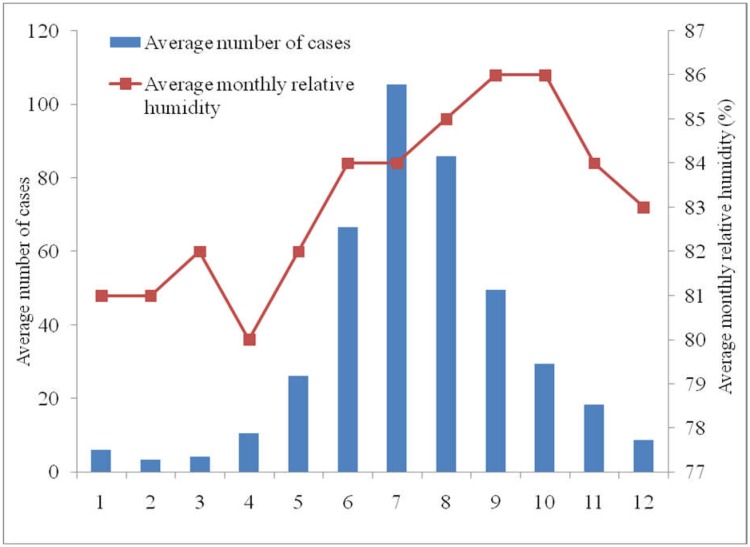
The correlation between DF/DHF cases and average monthly relative humidity (%) at Ba Tri District, 2004–2014.

The average relative humidity in Ba Tri District during the period 2004–2014 was 83.1% (SD = 1.99), with the highest relative humidity level being 86% (in August and September), and the lowest being 80% (in January and February). Figure 3 shows that DF/DHF cases increased in correlation with the relative humidity from April to July, but then decreased in the following months while the relative humidity continued to increase until October. There was a strong positive correlation between the total monthly DF/DHF cases and relative average humidity (*r* = 0.59).

There was a strong correlation between average monthly DF/DHF cases and average monthly climate variables (*r* = 0.69). However, when analyzing the trend (n = 132 months in 11 years), the correlation between DF/DHF cases and climate variability was quite weak *(r* = 0.30). Linear regression analysis showed that average monthly DF/DHF cases in Ba Tri District from 2004–2014 was strongly correlated with climate variables and vector density (*r* = 0.56 to 0.85).

**Figure 4.**
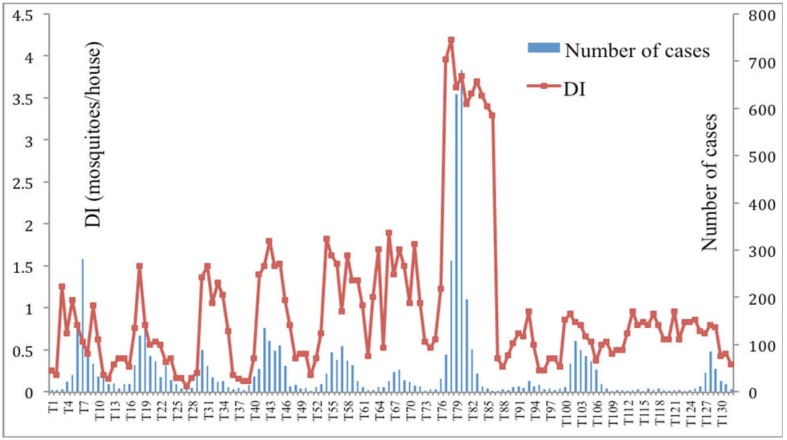
The correlation between number of monthly DF/DHF cases and monthly density index in Ba Tri District, 2014.

In 2014, the density index of *Aedes aegypti* (DI) exceeded the “safety” level, which is set at DI < 0.5 mosquito per household, from January to October. DI values increased approximately one month before DF/DHF cases started to increase. For the period 2004–2014, DI started to increase from March annually and followed by the increase in number of DF/DHF cases. In 2010, DI values varied from 0.5 to 4.2 mosquitoes/house, but in the previous years and from 2011–2014, the DI values were below 2.0 and the number of DF/DHF cases were also much lower than that in 2010 (Figure 4). There was a strong correlation between monthly DF/DHF cases with monthly DI values (*r* = 0.52).

**Figure 5.**
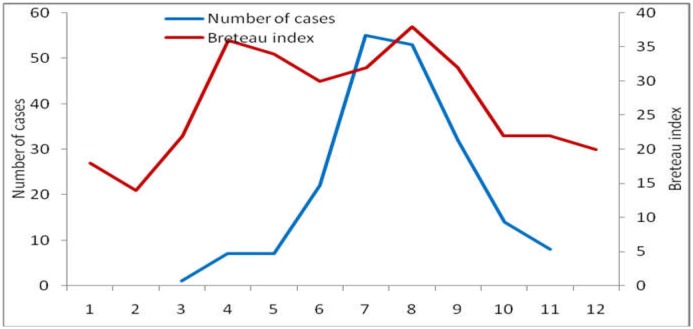
The correlation between number of monthly DF/DHF cases and monthly Breteau index in Ba Tri District, 2014.

For Breteau index, in 2014, the value started to increase from approximately 20 in March to nearly 40 in August. There were two peaks of BI value in the year 2014, which was 35 in April and 37 in August. There was a strong positive association between DF/DHF cases and BI values, with *r* = 0.66 (Figure 5). Multiple correlation showed a strong positive correlation between DF/DHF cases with DI and BI values (*r* = 0.68).

## 4. Discussion

The results of data analysis on vector density during the period 2004–2014 showed that the average monthly DF/DHF cases in Ba Tri District were positively correlated with DI, BI, and the peak in vector density occurred one month before the peak of DF/DHF cases. The BI values in the year 2014 in Ba Tri district were quite low in the dry season but increased in the rainy season and peaked at the value of 37 in August. This increasing trend in BI value from dry to rainy season was similar to the results reported in a study by Dang Ngoc Chanh et al. 2011, which showed that there was disparity in BI and other indices of four communes in Ben Tre Province during the rainy season and dry season [13]. Monthly average data for the period 2004–2014 showed strong correlation between monthly DF/DHF cases with DI (*r* = 0.82), BI (*r* = 0.81). Analyzing the trend from 2004 to 2014 (with the monthly data for 132 months) showed that monthly DF/DHF cases was quite strongly correlated with DI (*r* = 0.52) and moderately correlated with BI (*r* = 0.35). These results were similar with those reported in a study by Pham et al. in the Central Highland in the period of 2004–2010 and in Nghe An Province 2001–2010 [11],[14]. From 2011 to 2014, DI and BI values were lower than those of the period 2004–2010 and the total number of average monthly DF/DHF cases were also lower than the numbers reported before 2010. The *Aedes aegypti* density, BI, was critical in assessing the risk of DF/DHF. Epidemic and prevention measures, especially vector control measures must be implemented when BI was high.

The results also showed that DF/DHF cases occurred year round in Ba Tri District and started to increase from April to get the peak number in June-July. Average monthly DF/DHF cases were strongly positively correlated with average monthly rainfall (*r* = 0.70), average humidity (*r* = 0.59), and moderately correlated with average temperature (*r* = 0.37). Similar results were reported by Hoang et al. in Khanh Hoa City 2001–2010, by Toai in Can Tho City, 2001–2011, and by Truc in Bac Lieu Province in the period of 2006–2012 [15]–[17]. Another study in Metro Manila, Philippines showed a positive strong correlation between rainfall and DF/DHF cases from 1996 to 2005 [9]. Rainfall is important for different developmental stages of *Aedes* mosquitoes. In rainy months when the rain created more breeding sites and with favorable temperature, *Aedes aegypti* density increased. Temperature plays important role in transmitting DF/DHF disease because it affects the development, distribution, and blood sucking behavior of the *Aedes* mosquitoes. The relative humidity also correlated with temperature, rainfall and thus on *Aedes* mosquitos' development. Thus, this study and other studies conducted in other regions of Vietnam reported that DF/DHF increased in correlation with temperature, rainfall, relative humidity and vector density. DF/DHF shows strong seasonal variation and therefore, it is important to actively implement vector control measures at the beginning of the rainy season, approximately one to two months before the disease epidemic period as there was a lag time between climate conditions and disease outbreak. Limitations of the study include, the analysis only examining the correlation between the weather variability over time and DF/DHF rather than a causal relationship. Data in a longer period of time (e.g. two to three decades) and at provincial or national scales would provide more useful data for the analysis.

## 5. Conclusions and Recommendations

During the period from 2004 to 2014, there were strong positive correlations between the average montly DF/DHF cases with average monthly rainfall (*r* = 0.70), relative humidity (*r* = 0.59), *Aedes aegypti* density (*r* = 0.82), and BI (*r* = 0.81). There were moderate positive correlations between the average montly DF/DHF cases with average temperature (*r* = 0.37). The Ba Tri District Department of Preventive Medicine and commune health centers in Ba Tri District should monitor vector density, early diagnose DF/DHF cases at the beginning of rainy seasons starting from April, and apply active vector control measures to reduce the *Aedes* density and to prevent disease from developing and spreading in the following months. In addition, a larger study using data for all provinces and cities throughout Vietnam for a longer period (e.g. started from 1979 when data on DF/DHF and climate variables became available to 2015) should be undertaken to thoroughly describe the correlation between climate variability and DF/DHF cases as well as for modeling and to build projection model for the disease in the coming years based on climate data. This can play important role for active prevention of DF/DHF in Vietnam under the impacts of climate change and weather variability.
